# Next-generation Sequencing and Karyotype Analysis for the Diagnosis of Robertsonian Translocation Type Trisomy 13: A Case Report

**Published:** 2017-06

**Authors:** Jing SHA, Fumin LIU, Bei ZHANG, Yang HUANG, Qinglin ZHANG, Gao JUAN, Jingfang ZHAI

**Affiliations:** 1. Dept. of Obstetrics and Gynecology, Xuzhou Central Hospital, Xuzhou 221000, China; 2. The Affiliated Hospital of Xuzhou Medical College, Xuzhou 221000, China

**Keywords:** Robertsonian translocations, Trisomy 13, Karyotype, Next-generation sequencing

## Abstract

Trisomy 13 (Patau syndrome) is the third most common autosomal trisomy with a prevalence between 1 in 10,000 - 20,000 live births. Robertsonian translocations represent the largest number of chromosomal aberrations in human population with an incidence of 1.23 in 1000 live birth and translocation 13;14 is one of the most frequent Robertsonian translocations (approximately 75%). We sampled umbilical vein blood from a 27-yr-old woman whose ultrasonography findings revealed congenital heart defects, single ventricle, polycystic kidney, median cleft lip and palate and holoprosencephaly at gestational age of 23+6 weeks for karyotype and sequencing during intra-amniotic cavity injection of acrinol for labor induction. Next-generation sequencing indicated 47,XN,+13 and karyotype was identified as 46,XN,+13,rob (13;14). An unexpected problem becomes more and more obvious in human cytogenetics – it seems to become difficult to decide how and when to use the “molecular cytogenetics” or “traditional karyotype analysis”. Molecular cytogenetics, such as next-generation sequencing and array-based comparative genomic hybridization (array-CGH), can detect microdeletions and micro-duplications, but it cannot detect balanced translocations. For this case, we cannot find balanced translocations by Molecular cytogenetics. The purpose of this case is that molecular cytogenetics cannot replace the traditional karyotype analysis, but can serve as a useful complement for G-banding to be used in the clinical cytogenetic diagnosis.

## Introduction

Trisomy 13 syndrome, also known as Patau syndrome, is a congenital malformation syndrome caused by one extra chromosome 13 in group D chromosome (1). Neonatal incidence rate of trisomy 13 syndrome is about 1/10000 to 1/20000 (2). The main clinical features of 13 trisomy syndrome include serious defect in the central nervous system development, holoprosencephaly, light birth weight, severe mental retardation, small head, microphthalmos or anophthalmos, cleft lip and palate, cardiovascular malformation and polycystic kidney, etc. 50% children die within one month and 90% die within one year after born (3).

Robertsonian translocation’s incidence rate is 1.23/1000 (4), and the translocation between 13 and 14 accounts for about 75% of total Robertsonian translocations (5). Robertsonian translocations involve end-to-end fusion of two acrocentric chromosomes at or near the centromeres region, which results in the retaining of long arms of the two chromosomes and the lack of two short arms. As the lost short arms of the chromosomes are almost heterochromatin, the retaining long arms of the chromosomes contain almost all the genes in the chromosome. As a result, carriers of Robertsonian translocations are phenotypically normal but have increased risk of spontaneous abortions and livebirth of chromosomally unbalanced offspring (6). However, it is easy to bring about recurrent abortion and unbalanced aberration of fertility chromosome in offspring.

Robertsonian translocation type trisomy 13 can be detected by prenatal diagnosis technology, which inclue molecular cytogenetics and traditional karyotype analysis. Different diagnosis technologies have to be clearly distinguished and correctly applied.

## Case report

A 27-yr-old woman with first pregnancy was in the 25+3 week of pregnancy and her expected date of childbirth was April 25^th^, 2016. The baby’s fetal movement was normal. The threatened abortions, exposure to toxic substances and pregnancy-induced hypertension syndromes have never occurred. She self-reported that her age at menarche was 15, with menstrual cycle of 4-6/30 and normal menstrual volume. There was mild dysmenorrhea and no family history of genetic disease. Informed consent was taken from the patient and the hospital approved the study ethically.

On November 14^th^, 2015, the inspection blood of pre-pregnancy physical examination was sent to local Nanjing Gulou Hospital for pregnant meta-phase Down’s screening. As seen in Table 1, the fetus prenatal risk at trisomy 21 was 1:494, suggesting that the result of Down’s screening was critical risk. Non-invasive detection of DNA was recommended at this time but not performed. On December 31^st^, 2015 the fetal structural screening result at the local Hospital showed that the woman was in her 22-week+ of intrauterine pregnancy with single fetus, good fetal movement and whole forebrain. In addition, fetal heart malformation with single ventricle, fetal polycystic kidney, central fetal cleft lip and palate and missing vermis were suggested. After communication with her doctor, the patient decided to induce labour by intra amniotic injection and analysis of fetal chromosomal karyotype with umbilical vein puncture and next-generation sequencing detection.

**Table 1:** The result of Down's screening

Item	Result	MOM figure
AFP	28.8U/mL	0.81
Free-hCG	37ng/mL	2.26
21-trisomy risk	1:494	
18-trisomy risk	1:90919	
ONTD risk	low risk	

**Fig. 1:**
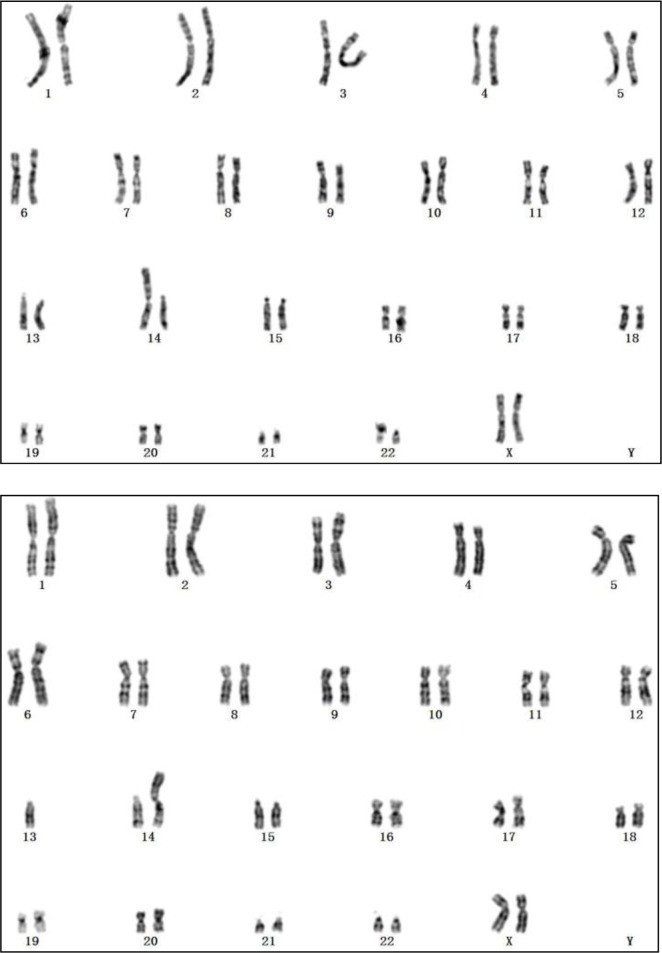
(A. Above) Fetal umbilical cord blood cells’ karyotype. (B. Down) The pregnant female karyotype

About 4 ml blood from the umbilical vein was extracted with guidance from ultrasound. Two ml was mixed evenly with peripheral blood culture medium and the medium was cultured in an incubator of 37 °C. After 72 h, conventional harvest chromosome specimens were prepared, and tested by G-banding. Two ml was sent to Hunan Gary Genetic Hospital for gene detection (Next-generation sequencing). Umbilical vein blood DNA was extracted with QIAamp DNA Blood Mini Kit, the sequencing library was established. High-throughput sequencing procedure was carried out on Illumina NextSeq500 platform. There were 30 metaphase cells were counted through radiology, 5 cells were selected to carry out karyotype analysis, showing that 13 and 14 chromosomes had translocations; At the same time, there was one more chromosome 13, which belonged to chromosomal translocation trisomy 13, with chromosome karyotype of 46, XN, +13, rob (13; 14) (Fig. 1A). Genetic testing results were 47, XN, +13. Suggestion of karyotype analysis was given to this couple. The pregnant female chromosome karyotype was 45, XX, rob (13; 14) (Fig. 1B) and the male chromosome karyotype was 46, XY.

## Discussion

The prenatal diagnosis of pregnant women is a corresponding examination clued on high-risk factors of fetal abnormalities. Cytogenetic or molecular biology detection is a key technique for prenatal diagnosis, but to get the fetal cells, we still rely on amniocentesis, chorionic villus, umbilical cord puncture and other invasive methods. Due to the relatively high risks and the high technical requirements, these methods are only suitable for high-risk pregnant women. Since the invention of chromosome banding technique in 1970, through continuous improvement, karyotype analysis technology has become a mature technology.

The gold standard for the diagnosis of chromosomal diseases uses chromosome karyotype to analyze the numbers and structures of chromosomes (7), which enables the prenatal diagnosis and prevention of some diseases. The karyotype analysis is still the most widely used method for prenatal diagnosis of fetal abnormalities among high-risk populations at present. With deeper understanding of disease and the improvements of medical technology, prenatal diagnosis technology has developed from cytogenetic chromosome level to molecular genetic single base site.

High-throughput DNA sequencing technology and gene chip technology have the advantages of high resolution, high sensibility, high throughput, rapidity and accuracy, etc. which challenges the conventional karyotype analysis. However, it can only detect the change of genomic copy number, such as deletion and duplication, making aberrations that cause no changes in copy number unable to detect, such as balanced translocation, inversion and so on. As this case of pregnant woman, the high-throughput DNA sequencing result was 47, XN, +13 and the karyotype was 46, XN, +13, rob (13; 14). We detected the chromosomes of this couple in order to determine if the fetal translocation was new or genetic. The result showed the woman as a carrier of Robertson translocation, and she was told that the individual germ cell meiosis can produce 6 gametes, leading to zygotes with 1/6 normal type, 1/6 translocation type and others for monosome or trisome (8). Currently we can use the preimplantation genetical diagnosis (PGD) technique to screen for normal embryos (9–11), in order to avoid mental and physical trauma caused by repeated spontaneous abortion. If the woman had not received the traditional chromosome karyotype analysis technology, translocation would be missed.

## Conclusion

Molecular karyotype techniques (e.g. high-throughput DNA sequencing or gene chip technology) can detect microdeletions and repetitions that cannot be detected by karyotyping, and may be used as a complement to conventional analysis techniques for cell genetic diagnosis.

## Ethical considerations

Ethical issues (Including plagiarism, informed consent, misconduct, data fabrication and/or falsification, double publication and/or submission, redundancy, etc.) have been completely observed by the authors.

## References

[1] Patau K Smith DW Therman E (1960). Multiple congenital anomaly caused by an extra autosome. Lancet, 1(7128):790–793. 14430807 10.1016/s0140-6736(60)90676-0

[2] Mbuyi-Musanzayi S Lumaka A Yogolelo Asani B (2014). Preaxial polydactyly of the foot: variable expression of trisomy 13 in a case from central Africa. Case Rep Genet, 2014: 365031. 25254124 10.1155/2014/365031 PMC4164427

[3] Karabel M Yolbas I Kelekci S (2013). A newborn with trisomy 13 who had tetralogy of Fallot and metopic synostosis: Case report. Hippokratia, 17(3):268–270. 24470740 PMC3872466

[4] Nielsen J Wohlert M (1991). Chromosomes abnormalities found among 34910 newborn children:results from 13 year incidence study in Arhus, Denmark. Hum Genet, 87(1):81–83. 2037286 10.1007/BF01213097

[5] Scriven PN Flinter FA Braude PR (2001). Robertsonian translocations—reproductive risks and indications for preimplantation genetic diagnosis. Hum Reprod, 16(11):2267–2273. 11679502 10.1093/humrep/16.11.2267

[6] Choi BH Kim UH Lee KS (2013). Various endocrine disorders in children with t(13;14)(q10;q10) Robertsonian translocation. Ann Pediatr Endocrinol Metab, 18(3):111–115. 24904863 10.6065/apem.2013.18.3.111 PMC4027073

[7] Comas C Echevarria M Carrera M (2010). Rapid aneuploidy testing versus traditional karyotyping in amniocentesis for certain referral indications. J Matern Fetal Neonatal Med, 23(9):949–955. 20718579 10.3109/14767050903334893

[8] Li CH Zhang XM Li YG (2004). [Preimplantation genetic diagnosis fora patient with Robertsonian translocation]. Zhonghua Yi Xue Yi Chuan Xue Za Zhi, 21(5):488–490. 15476178

[9] Yilmaz A Zhang XY Chung JT (2012). Chromosome segregation analysis in human embryos obtained from couples involving male carries of reciprocal of Robertsonian translocation. Plos One, 7:e46046. 23029381 10.1371/journal.pone.0046046 PMC3459837

[10] Bernicot I Schneirob A Mace A (2012). Analysis using fish of sperm and embryos from two carriers of rare rob (13;21)and rob(15;22) Robertsonian translocation unrob-going PGD. Eur J Med Genet, 55:245–251. 22406402 10.1016/j.ejmg.2012.02.003

[11] Lukaszuk K Pukszta S Ochman K (2015). Healthy Baby Born to a Robertsonian Translocation Carrier following Next-Generation Sequencing-Based Preimplantation Genetic Diagnosis: A Case Report. AJP Rep, 5(2):e172–e175. 26495179 10.1055/s-0035-1558402 PMC4603858

